# Evaluation the Solubility and the Porosity of the Nano Fast Cement Comparing to the Mineral Trioxide Aggregate: An *in vitro* Study

**DOI:** 10.30476/DENTJODS.2021.92684.1668

**Published:** 2023-03

**Authors:** Fariborz Moazami, Zahra Rajabzadeh, Habib Daneshmanesh, Yasmin Ghahramani

**Affiliations:** 1 Dept. of Endodontic, School of Dentistry, Shiraz University of Medical Sciences, Shiraz, Iran; 2 Dept. of Chemical Engineering, Shiraz University of Engineering, Shiraz, Iran

**Keywords:** Solubility, Porosity, Cement, Endodontics, MTA

## Abstract

**Statement of the Problem:**

New calcium silicate base cements are introduced as root repair materials in order to defeat the problems of early root repair materials. Their mechanical properties such as solubility and porosity should be concerned

**Purpose:**

This study was conducted to evaluate the solubility and porosity of the NanoFastCement (NFC) as a new calcium silicate base cement comparing to mineral trioxide aggregate (MTA).

**Materials and Method:**

In this in vitro study, scanning electron microscope (SEM) was used to evaluate the porosity at five different magnifications (200×, 1000×, 4000×, 6000× and 10000×) in secondary backscattered electron mode. All analyses were performed at 20kV. The obtained images were subjected to qualitative evaluation regarding the porosity. Solubility was determined following the international standards organization (ISO) 6876 method. Twelve specimens in specially fabricated stainless steel ring molds were weighed, initially and after 24 hour and 28 days of immersion in distilled water. Each weight was measured three times to record the average weight. Solubility was measured by calculating the difference of the initial and final weight.

**Results:**

Solubility of the NFC in comparison with MTA showed no statistical difference (*p* Value > 0.05) after one day and 28 days. NFC acted like MTA and showed an acceptable solubility value at exposure time intervals. In both groups, solubility increased as time went on (*p* Value<0.05). The porosity of NFC was comparable to MTA, and NFC presented a less porous and a slightly smoother surface compared to MTA.

**Conclusion:**

NFC has similar solubility and porosity to Proroot MTA. Therefore, it can be a good, more available and less expensive substitute for MTA.

## Introduction

Root repair cements that are commonly used in endodontics should have appropriate physicochemical properties. They are supposed to provide a proper seal to avoid bacterial infiltration and endotoxins from the root canal to the periodontium ADDIN EN.CITE ADDIN EN.CITE.DATA [ 1 ]. Along with other necessary assets, they must be biocompatible, dimensionally established, non-absorbable, and not influenced by moisture of environment [ 2 - 3 ]. Besides, their low solubility is important [ 4 - 5 ] since it prevents the dissolution of the materials in the body fluid, which subsequently evades material leakage [ 4 , 6 ].

Solubility is a crucial factor in evaluating the dental materials. Since the long-term seal of these biomaterials is highly desirable in clinical practice, low solubility can avoid the fluid leakage from both the oral cavity and the periapical area [ 7 - 8 ]. One of the important characteristics of root repair materials is lack of solubility [ 9 ]. Several dental materials including root-end filling materials have been broadly studies for solubility. Mineral trioxide aggregate (MTA), a hydrophilic combination of tri- and/or dicalcium silicates which is set by addition of water and forming calcium silicate hydrate gel ADDIN EN.CITE ADDIN EN.CITE.DATA [ 10 - 12 ], has no or low solubility [ 7 , 13 - 14 ].

Studies demonstrated that calcium silicate cements in the presence of phosphate-containing fluids, could induce calcium phosphate deposition on their surface. However, it is unclear if crystal formations would influence the solubility or not [ 10 , 15 - 16 ].

In a study, although in the acceptable range, Biodentine was significantly more soluble than ProRoot MTA in all periods [ 8 , 17 ]. In a study by Grech *et al*. [ 18 ], the solubility of Bioaggregate, Biodentine, intermediate restorative material (IRM), and tricalcium silicate cement was assessed for 28 days. The results indicated there was no significant difference between the materials. In order to compare the solubility of Angelus MTA versus Portland cement (PC), Bodanezi *et al*. [ 19 ] have shown that Angelus MTA exhibits over 3% weight loss in the first 24h after mixing, which according to the international standard organization(ISO) is not acceptable.

Another crucial characteristic of root repair material is the porosity affecting the physical properties and their environmental behavior [ 20 ]. A porous material is prone to leakage which leads to treatment failure [ 6 , 20 ]. Porosity and solubility are highly effective factors in the materials stability, integrity, and durability [ 21 ]. 

The porosity is assessed by evaluation the size and pores allocation in the polished material surface; this evaluation is not quite accurate because of its qualitative nature [ 22 ]. The porosity of endodontic materials is evaluated using an optical microscope, micro-CT, or scanning electron microscope (SEM). Evaluation via SEM is an improved option because it is a more accurate method than the optical microscope [ 22 - 24 ].

 Studies have evaluated MTA porosity [ 23 - 24 ]. Its porosity is related to some factors including the water powder ratio, air entrapment during the mixing, or the environmental pH value [ 5 , 23 - 26 ]. In addition, the porosity may be associated with solubility [ 27 - 29 ].

NanoFastCement (NFC) is a new calcium silicate-based cement recently introduced to endodontics as a root repair material [ 30 - 31 ]. The milling machine reduces the particle size, and its mechanical properties are significantly improved with adding up a multi-walled carbon nanotube [ 30 - 31 ]. It is suggested that the addition of multi-walled carbon nanotubes can decrease the porosity of Portland cement pastes, which can increase the mechanical properties [ 31 ]. This material has short setting time and high strength. It is also a biocompatible material with similar antimicrobial and antifungal properties and the same color change as MTA [ 32 - 33 ].

The present study was conducted to evaluate the solubility and porosity of NFC comparing to MTA. 

## Materials and Method

In order to assess the solubility, standard samples were fabricated. The samples were weighed before and after immersion in distilled water according to the international standards organization (ISO) 6876:2002 [ 8 ].

Twelve individually fabricated stainless steel ring molds with an internal diameter of 20±0.1mm and a height of 1.5±0.1mm were used for the preparation of samples in two groups. The first group (n=6) for Proroot MTA, and the second group (n=6) for Nano Fast Cement. The molds were cleaned using acetone in an ultrasound bath for 15 min, then left to dry for 30min. The same operator mixed the restorative materials in line with manufacturer’s instructions in manual. 

The ring molds were located on a glass slab and filled to excess with the mixed materials. Samples were left to set in an incubator maintained at 37°C. After 24 hours, samples were exposed to air for 15 minutes. The samples were then weighed three times to register the average reading. This weight was defined as the initial weight (IW) of the samples. After that, distilled water was added to the samples. They were transferred to the incubator until they were evaluated after 1 day (24 hours) and 28 days using the same method. Each ring was separately weighed and recorded as final weight (FW). The solubility of each sample was calculated by using the subsequent equation [ 6 ].

Solubility = (FW –IW) × 100

In order to assess the surface morphology, the materials were inserted into cylindrical molds with 6mm width and 12mm height. Specimens were reserved in an oven at 37°C and immersed in distilled water for 28 days. Afterwards, the test specimens were dried with absorbent paper and kept in a vacuum desiccator containing silica for 24 hours. Then, the samples were immersed in resin and polished with the automatic polishing machine. The samples were dried and placed on stubs, coated with carbon for scanning electron microscope (SEM) imaging at five magnifications (200x, 1000x, 4000x, 6000x and 10000) in Secondary backscattered electron mode. All analyses were performed at 20kV. The images obtained were subjected to qualitative evaluation regarding the porosity [ 6 ].

The data were analyzed using statistical package for social sciences (SPSS) software, v.11.0 (SPSS Inc, Chicago, IL) and subjected to independent (student) T-test with the statistical difference set at *p*< 0.05.

## Results

**Figure 1:**
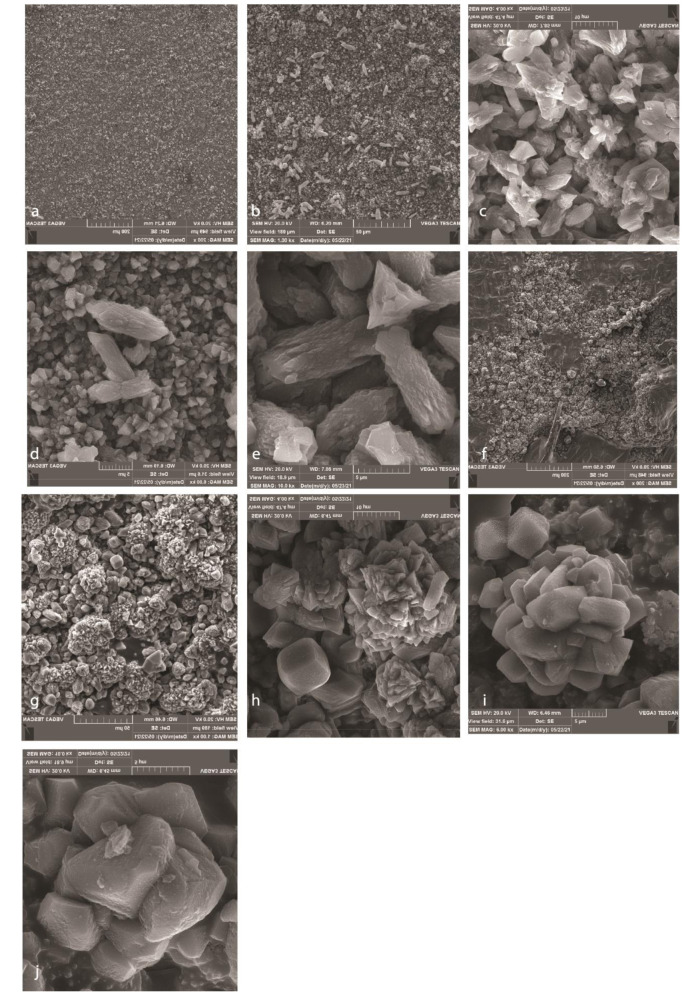
Scanning electron microscope (SEM) images of NanoFastCement (NFC) (a,b,c,d,e) and mineral trioxide aggregate (MTA) (f,g,h,I,j) showing the magnification of 200,1000,4000, 6000, and 10000 respectively. In similar magnifications, the surface of NFC is smoother with fewer pores in comparison to MTA

The solubility test results with statistical comparison of mean and standard deviation between the groups at different time intervals are presented in Table 1. NFC solubility was 2.7% after 24h and 3.04% after 28 days. Solubility of NFC in comparison with MTA after 24hours and 28 days had no statistical differences (*p*> 0.05). NFC showed an acceptable solubility value at exposure time intervals. However, both groups showed increased solubility as the time went on to the second time interval (*p*= 0.023). SEM evaluation of NFC showed a smoother surface than MTA. NFC had smaller particles, and fewer and smaller pores than MTA. Particles of NFC were sharp and pointed in comparison to MTA particles with cauliflower figure (Figure 1). Figure 1 shows the porosity of MTA and NFC with different magnifications of SEM.

**Table 1:** (mean± SD) solubility of MTA and NFC after 24 hours and 28 days

Time	After 24h mean± SD	After 28days mean± SD
Group		
NFC	2.1% ± 0.37	3.04% ± 0.35
MTA	2.27%± 0.0	3.51% ± 0.03
*p* Value^*^	0.18	0.133

MTA: mineral trioxide aggregate

NFC: NanoFastCement

## Discussion

Solubility of cement can affect its structural integrity, dimensional stability, durability and subsequently its clinical success in dental practice [ 34 - 35 ]. The amount of weight loss determines this property after immersion in a solution [ 36 ]. Factors such as immersion time, chemical composition, size of valuable material, and the chemistry of the solvent affect solubility [ 37 ].

Originally, NFC was developed by modification of MTA structure, which resulted in its short setting time and high strength. It is a biocompatible material with similar antimicrobial and antifungal properties and the same color change as MTA [ 30 - 31 ]. In this study, we found that its solubility was similar to MTA. NFC solubility was 2.7% % after 24h and 3.04% after 28 days. Therefore, it fulfilled the requirements of ISO 6876:2001 like ProRoot MTA.

Among different root repair materials, MTA is the gold standard due to its acceptable properties the solubility of MTA is debated among investigators. However, most of the studies have reported the solubility of this material to be negligible [ 7 , 13 - 14 , 38 - 39 ]. However, relatively high solubility of this material has been reported in one long-term investigation [ 16 ]. Regarding the *in vitro* studies, ProRoot MTA can be considered as practically insoluble root-end filling material [ 7 , 13 - 14 , 35 ]. The current study showed 2.27% weight loss of MTA samples similar to other studies [ 13 - 14 , 35 ]. It was found that both Biodentine and ProRoot MTA fulfilled the requirements of ISO 6876:2001 and showed solubility less than 3% after 24 hours [ 8 , 40 ]; the evaluation of NFC solubility showed similar results in the current study.

Generally, the porosity appears because of developed spaces in the non-hydrated cement [ 22 ]. The porosity and solubility of materials can have a substantial impact on their stability, integrity, and durability [ 38 ]. The porosity can be verified by visually evaluation of the size and allocation of pores on the polished surface of the cement, though this qualitative type of assessment is not precise [ 22 ]. Porosity is usually due to incorporating microscopic air bubbles during the mixing operation [ 7 , 39 ]. However, an amorphous porous and capillary structure observed with SEM could be another important cause of the materials porosity [ 39 ].

SEM images of set MTA and NFC are shown in figure 1; NFC porosity was less than MTA. SEM images of NFC revealed that the surface of the NFC was less porous and smoother than MTA. Its crystals were sharp, pointed and like a prism; whereas MTA crystals were round and like cauliflower. It might be because of the size of the particles, which are smaller in NFC, and crystallization of each material, or changes in chemical structure of them. 

The zirconia particles can be observed on the surface of MTA as white dots, which have a smaller in size in NFC. This smoothness is probably because of the smaller particle size of NFC due to the milling procedure [ 40 ]. 

It is acquainted that insolubility is a desirable property for root-end filling materials in dentistry [ 9 ]. The current study was conducted to evaluate the materials solubility after 24h and 28-days because longer experimental intervals are important when the behavior of a material is being analyzed.

In clinical situations, only part of the root-end filling materials is in the direct contact with the aqueous environment (*i.e.,* periapical tissues) [ 26 ], but the whole specimen was in contact with the aqueous environment in the present study. Therefore, the amount of solubility might be less than clinical situation in this study. On the other hand, solubility may be even more than our achieved result, because it was an *in vitro* study and we used set material in the present study but in clinical situation, the substance should set in oral condition, which is an aqueous environment, and is more prone to dissolution. Therefore, *in vivo* studies are needed in future to evaluate these properties accurately.

## Conclusion

NFC has similar solubility and porosity to Proroot MTA, a gold standard in root repair materials. Therefore, it can become an excellent, more available, and less expensive substitute for MTA. 

## Conflict of Interest

The authors declare that they have no conflict of interest.
